# Expanding on “Analyzing Patterns in Anesthesiology Residents' Exam Performance Using Data Mining Techniques”

**DOI:** 10.5812/aapm-159478

**Published:** 2025-03-17

**Authors:** Seyed Mohammad Seyed Alshohadaei, Fereshteh Baghizadeh

**Affiliations:** 1 Shahid Beheshti University of Medical Sciences, Tehran, Iran

**Keywords:** Anesthesiology Education, Data Mining, Clinical Reasoning, Educational Measurement, Critical Thinking, Performance Analysis

*Dear Editor,*

I thoroughly enjoyed reading the article "Analyzing Patterns in Anesthesiology Residents' Exam Performance Using Data Mining Techniques" and commend the authors for their innovative use of data mining to analyze trends and performance predictors in anesthesiology education. This work underscores the potential of data-driven insights to revolutionize resident training and curriculum design.

One aspect that could further enrich this research is the inclusion of clinical reasoning tests (CRT) as a tool to assess critical thinking and decision-making skills. Clinical reasoning is a cornerstone of anesthesiology, where prompt and accurate decisions directly impact patient outcomes. Incorporating CRT metrics in future analyses could provide a deeper understanding of how residents translate theoretical knowledge into practical application under pressure.

Moreover, CRT has been shown to align closely with real-world challenges in clinical practice. Studies suggest that reasoning-based assessments can identify specific areas where residents struggle, such as prioritization in emergency scenarios or adapting to rapidly evolving situations (1-3). Integrating CRT into data mining frameworks presents several key opportunities:

(1) Correlation between reasoning and exam performance: Exploring whether higher clinical reasoning scores predict stronger performance in knowledge-based exams or improved patient outcomes.

(2) Targeted interventions: Using CRT results to design focused training modules, such as simulation-based learning or scenario-driven workshops, that address specific reasoning deficiencies.

(3) Feedback loops for curriculum development: Linking CRT data with exam results to refine educational strategies and ensure alignment with real-world anesthesiology demands.

Additionally, incorporating reasoning-focused assessments into data-driven studies could provide insights into residents’ preparedness for independent practice, particularly in critical care settings where sound judgment is essential (4, 5).

This article lays a strong foundation for integrating educational data, and I encourage future research to explore clinical reasoning as a complementary dimension in performance analyses. The combination of reasoning assessments with data mining techniques has the potential to enhance anesthesiology training and improve resident competency.

Thank you for this valuable contribution to the field. I look forward to seeing how these insights evolve in future studies.

## References

[1] Omega A, Wijaya Ramlan AA, Soenarto RF, Heriwardito A, Sugiarto A (2022). Assessing clinical reasoning in airway related cases among anesthesiology fellow residents using Script Concordance Test (SCT).. Med Educ Online..

[2] Amini M, Moghadami M, Kojuri J, Abbasi H, Abadi AA, Molaee NA (2011). An innovative method to assess clinical reasoning skills: Clinical reasoning tests in the second national medical science Olympiad in Iran.. BMC Res Notes..

[3] Sadeghi A, Ali Asgari A, Moulaei N, Mohammadkarimi V, Delavari S, Amini M (2019). Combination of different clinical reasoning tests in a national exam.. J Adv Med Educ Prof..

[4] Ramier M, Clavier T, Allard E, Lambert M, Dureuil B, Compere V (2024). Examining the impact of sleep deprivation on medical reasoning's performance among anaesthesiology residents and doctors: a prospective study.. BMC Anesthesiol..

[5] Bengayed K, Akermi S, Amari Y, Chouigui R, Haddad F (2024). Assessment of Clinical Reasoning in Healthcare Students via the Script Concordance Test: Situations in Anesthesia and Intensive Care.. Tunis Med..

